# Personalizing Patient Education for Pancreatic Cancer Patients Receiving Multidisciplinary Care with Integration of Novel Digital Tools

**DOI:** 10.3390/healthcare13151929

**Published:** 2025-08-07

**Authors:** Nicole Nardella, Matt Adams, Adrianna Oraiqat, Brian D. Gonzalez, Corinne Thomas, Sarah Goodchild, Sonia Adamson, Maria Sandoval, Jessica Frakes, Russell F. Palm, Carrie Stricker, Joe Herman, Pamela Hodul, Sarah Krüg, Sarah Hoffe

**Affiliations:** 1 Department of Gastrointestinal Cancer, Moffitt Cancer Center, Tampa, FL 33612, USA; adrianna.oraiqat@moffitt.org (A.O.); pamela.hodul@moffitt.org (P.H.); 2 Desert Regional Medical Center, Palm Springs, CA 92262, USA; mattadams3377@gmail.com; 3 Department of Health Outcomes and Behavior, Moffitt Cancer Center, Tampa, FL 33612, USA; brian.gonzalez@moffitt.org; 4 University of South Florida, Tampa, FL 33620, USA; cthomas48@wisc.edu (C.T.); sarahg06062@gmail.com (S.G.); 5 Department of Radiation Oncology, Moffitt Cancer Center, Tampa, FL 33612, USA; sonia.adamson@moffitt.org (S.A.); maria.sandoval@moffitt.org (M.S.); jessica.frakes@moffitt.org (J.F.); sarah.hoffe@moffitt.org (S.H.); 6 Department of Radiation Oncology, The Ohio State University, Comprehensive Cancer Center, Columbus, OH 43210, USA; russell.palm@osumc.edu; 7 Canopy Cancer Collective, Scotts Valley, CA 95066, USA; carrie.stricker@1440.org (C.S.); joe.herman@1440.org (J.H.); 8 CANCER101: Health Collaboratory, New York, NY 10163, USA; sarahkrug@cancer101.org

**Keywords:** patient education, learning preferences, personalized care, digital health, emotional assessment, shared decision making, distress, patient experience, empathy, patient satisfaction

## Abstract

**Background/Objectives:** Pancreatic cancer (PC) is a diagnosis with a poor prognosis which can be associated with significant distress and may hinder a patient’s ability to understand treatment details. Educating patients based on their learning preferences (LPs) and emotions may allow for personalized, enhanced care. **Methods:** This prospective project enrolled patients with non-metastatic PC. Phase 1 utilized the Learning Preference Barometer (LPB) and Emotional Journey Barometer (EJB), which are digital instruments co-designed by CANCER101 (C101) and the Health Collaboratory, to assess patient LPs and emotional states. Phase 2 provided information prescriptions aligned with LPs through C101’s Prescription to Learn^®^ (P2L) platform. Collected data included demographics, treatment, LPs (auditory, kinesthetic, linguistic, visual), patient engagement with P2L, and patient emotional states with qualitative verbal validation. Descriptive variables were used to report outcomes. **Results:** Primary LPs in the 47 participating patients were as follows: linguistic 45%, visual 34%, auditory 11%, and kinesthetic 9%, with secondary preferences in the majority (53%). Those patients (66%) who accessed P2L had linguistic and visual preferences; the majority accessed 1- 2 resources out of the 25 provided. Resources accessed aligned to 88% of patient LPs. The majority of patients (60%) initiated treatment prior to initial EJB, and 40% were treatment naive. Common baseline emotions were optimistic (47% vs. 36%, respectively), satisfied (11% vs. 25%), acceptance (11% vs. 11%), and overwhelmed (5% vs. 11%). **Conclusions:** Assessing LPs and emotional state allows for personalized patient education and clinical encounters for PC patients. Future work includes examining the effects of personalized approaches on patient satisfaction, decision-making, health outcomes, and the overall patient–clinician relationship.

## 1. Introduction

Distress, confusion, uncertainty, and anxiety often accompany a new cancer diagnosis. Despite treatment advances, up to 60% of patients still experience significant levels of distress during their treatment journey [1]. Distress is an all-encompassing facet of the patient experience, composed of psychological, social, spiritual, and physical factors that can interfere with and impede a patient’s understanding and treatment [2]. This distress can lead to a reduction in quality of life and overall decreased survival [3,4]. The patient–clinician relationship is a complex psychosocial interplay of connection, partnership, trust, effective communication/education, empathy, and emotional support, which can have profound implications on clinical care, including health outcomes, and overall experience. Effective clinician communication can help manage patients’ emotions, facilitate comprehension of medical information, and allow for better identification of patients’ needs, perceptions, and expectations [5].

Although standards of care for treatment are well documented, optimal patient education and practice varies by specialty, clinician, and location. Patients/care partners may be overwhelmed with new information and comprehension can be complicated by unfamiliar terminology. Although patients report a need for more information, delivery at one time can be overwhelming [6]. Research has shown that the average patient can forget 40–80% of the information communicated by clinicians. Of the information they do recall, approximately half of it is remembered correctly [7,8]. One of the most promising education reforms is personalizing education through patient learning preferences (LP), which can improve health literacy, leading to better retention of information [9,10]. The term “learning preference” has been coined to acknowledge that how patients learn is situational, and can differ based on time, scenario, and location compared to the term “learning style” which indicates a fixed modality [11,12,13,14]. However, cancer patients’ baseline LP and emotional states are not well documented at the point of care (POC). CANCER101 (C101), a non-profit, patient advocacy organization, and the Health Collaboratory, an innovation hub anchored on co-design, partnered with patients, care partners, clinicians, and other organizations to co-design a series of instruments we evaluated to address these gaps. Through this quality improvement (QI) pilot project, we aimed to determine if we could integrate learning preference and emotional assessments to personalize patient education during routine care for patients with pancreatic cancer (PC).

## 2. Materials and Methods

This IRB-exempt, QI prospective pilot project was conducted in the Gastrointestinal Oncology Program. Eligibility criteria included: (a) diagnosed with non-metastatic PC, (b) scheduled to receive chemotherapy, radiation, and surgery at Moffitt Cancer Center (i.e., to maximize the variety of educational resources accessed), and (c) patients at least 18 years of age [15]. Exclusion criteria included patients with metastatic disease after enrollment, as their educational needs would vary from those of patients with non-metastatic disease. Eligible patients had previously consented on our IRB approved institutional lifetime biorepository program called Total Cancer Care.

The project incorporated several digital instruments. The first, the Learning Preference Barometer (LPB), assesses LPs through a series of multiple-choice questions based on real-world experiences to assess preferred methods of receiving, understanding, processing, and exchanging information. To create this, a participatory co-design process was utilized to partner with 322 patients and care partners to ensure that the final instrument aligned with needs, preferences, and individual experiences. A landscape assessment was conducted to understand current methods in determining LPs, which were then evaluated by patients/care partners across various domains, including patient-centricity and user experience. These insights were used to build the LPB, which was then tested against other validated tools to ensure that the final output was consistent (Appendix A.1).

The second tool, the Emotional Journey Barometer (EJB), was co-designed with 94 patients and care partners to capture the main emotions experienced across their health journey. To initially map the most prevalent emotions, qualitative, semi-structured interviews were conducted utilizing an ethnographic approach to allow patients/care partners to share their health stories and co-produce journey maps. A participatory storytelling technique was used to provide a safe zone for information exchange with a deeper dive into emotions experienced and rationale across phases of the care journey. A total of 32 different emotions were identified across the patient pathway with 11 outliers. Results showed 21 emotions were the most prevalent emotions experienced and used to co-design the EJB, which was then validated with 88 patients/care partners across different points in their health journey. An abbreviated version of the EJB was used for this project (Appendix A.2).

The third, Prescription to Learn^®^ (P2L), was created to guide cancer patients through the information overload they often face throughout the care journey, aligning them with the right resources at the right time to help them better understand their condition and make informed decisions in partnership with their healthcare team. P2L was co-designed with 125 patients and care partners, along with other organizations, using a rigorous curation and review process to map the most effective resources from credible sources. P2L provides the opportunity for patients/care partners to personalize their search for education by disease state, phase in the journey, medium of choice, and LP. Clinicians can also prescribe personalized resources to patients/care partners (Appendix A.3).

This project consisted of two phases: Phase I included assessment of LPs and emotions; Phase II included targeted personalized education based on output from Phase I. Phase I utilized the LPB and EJB to assess patient LP (at baseline, defined as project enrollment date) and emotional state (at baseline and follow-ups). Patients were assigned a project ID to anonymize their data. The research project coordinator conducted the LPB and EJB assessments during regularly scheduled clinic visits. In Phase II, patients were provided information prescriptions through the P2L platform. Project participants received a physical C101 Planner, personalized with institutional PC-specific education. 

After completion of the LPB, LP results were displayed, and patients were given a primary LP. A secondary LP was assigned if there were at least 5 responses in an additional LP. For the EJB, patients indicated the emotion they were feeling at that moment by selecting an emoji; a free response box was available for those who did not see an appropriate emoji. Patients were asked to verbally explain why they selected the emotion, and responses were audio recorded by the project coordinator to allow for natural conversation. Phase I assessment took approximately 6 min to complete.

Phase II leveraged the P2L platform, which included 25 institutional educational resources. Each patient received an access code (project ID) to P2L with instructions on how to utilize the platform and personalize educational resources by phase of journey, LP, and medium of choice. The project coordinator provided a demonstration on utilizing P2L to ensure patients could access independently.

Additional patient data was collected via chart review. Patient demographics included age, gender, Florida regionalization vs. out of state, Area Deprivation Index (ADI) scores (state and national), and Charlson Comorbidity Index (CCI) scores (standard and age adjusted). ADI scores are calculated using socioeconomic conditions, with high scores indicating greater deprivation. CCI is a validated instrument to measure mortality rate and disease burden based on comorbidities, with age adjustment of 1 point per decade of age over 40 years. Sociodemographic data was collected related to financial concerns (unemployment, insurance, etc.), dependent children, lifestyle concerns (lives alone, transportation issues, lack of support), education (high school/GED or trade school), and insurance/employee disability. Patient LPs and barriers as well as learning motivation data were recorded from standard of care nursing note assessments, collected at the first clinic consultation. Additionally, performance status scores (Eastern Cooperative Oncology Group (ECOG)) and the National Comprehensive Cancer Network Distress Thermometer (NCCN-DT) scores were collected during regular clinical practice, prior to EJB assessment.

Descriptive variables were used to report outcomes. Measures of central tendency, including means, medians, ranges, and percentages/probabilities were analyzed. 

The sample size for this QI project was selected based on prior literature indicating that a sample of ≥25 would be sufficient for achieving saturation, or the point at which no new relevant information would be obtained from additional qualitative interviews [16]. We planned to enroll 50 participants to account for risk of development of metastatic disease, non-adherence, and loss to follow-up.

## 3. Results

The project included 50 adult (>18 years), non-metastatic pancreatic cancer (PC) patients. Within one month of enrollment, 3 patients were found to have metastatic disease, and therefore excluded from analysis. The patient population was 53% male (n = 25) and 47% female (n = 22), with a median age of 70 years old (range of 52–88). ADI state and national scores were recorded, with medians of 7 (1–10) and 56 (4–98), respectively. CCI scores were calculated to include total and age adjusted scores. The median total score was 3 (2–7), while the age adjusted was 6 (4–11). See Table 1 for demographics.

### 3.1. Learning Preference Barometer

The most common LP was linguistic (45%, n = 21), followed by visual (34%, n = 16), auditory (11%, n = 5), and kinesthetic (9%, n = 4). Secondary LPs were present in 53% of patients (n = 25), with a correlation between linguistic and auditory learners (Table 2).

The male patients’ LP results were: 44% visual (n = 11/25), 28% linguistic (n = 7/25), 12% auditory (n = 3/25), 12% kinesthetic (n = 3/25), and 4% auditory/visual combination (n = 1). The female patients’ LP results were: 64% linguistic (n = 14/22), 23% visual (n = 5/22), 9% auditory (n = 2/22), and 5% kinesthetic (n = 1/22). LPs were consistent across age groups; however, the 50–60 and 81–90 sub-cohorts had stronger visual preferences and no kinesthetic preferences. Primary LPs were viewed by ADI scores, with most visual patients having higher ADI scores (Appendix A.4).

Patient LPs were documented through institutional subjective nursing assessments for 81% of patients (n = 38). Results are displayed in Table 3. The majority (76%) of patients had reading, verbal or video/visual LP documented, demonstrating the lack of specificity with this assessment. 

### 3.2. Prescription to Learn^®^

Patients were given access codes to P2L upon completion of the LPB. Of the cohort, 66% (n = 31) accessed P2L. The median age of patients accessing P2L was 70 (range 52–86). Table 4 displays further breakdown by age and gender. Of the patients who had online access, 45% were male, and 54% were female. Results demonstrated that patients with linguistic (48%) and visual (32%) primary LPs accessed P2L more frequently. The number of resources patients accessed was as follows: 48% (n = 15) one resource, 42% (n = 13) 2 resources, 3% (n = 1) 3 resources, 3% (n = 1) 6 resources, and 3% (n = 1) 8 resources. Most resources were accessed at least once (68%, n = 17/25); 41% were accessed once (n = 7/17), 35% were accessed twice (n = 6/17), and 24% were accessed more than 2 times (n = 4/17). Note that 88% of the resources accessed (n = 15/17) had LP results that correlated with the patients’ LPB results.

Additionally, analysis of P2L access showed access by patients with social determinants to be relatively high: 83% with dependent children, 50% with disabilities, 43% with educational barriers, 78% with financial concerns, and 57% with lifestyle concerns.

### 3.3. Emotional Journey Barometer

All patients completed the EJB at project enrollment. Sample EJB responses and patient explanations are stated in Table 5. The most reported emotions at enrollment were optimistic (43%), satisfied (19%), and acceptance (11%). When compared to ADI scoring, results demonstrate patients with higher ADI scores were more likely to select optimistic and satisfied emojis (Appendix A.4). Based on social determinants, 14% of patients with educational barriers indicated confusion and 11% of patients with financial concerns indicated stress. Patients with lifestyle concerns experienced a larger spectrum of emojis with 43% selecting optimistic, 22% satisfied, and 7% acceptance, angry, anxious, overwhelmed, or stress.

Based on willingness and clinical availability, 45% (n = 21) of patients were able to complete a second EJB at a subsequent clinic visit. The most common responses were: 33% acceptance, 33% optimistic, 14% satisfied, and 10% happy. Patients with higher ADI scores tended to indicate acceptance (Appendix A.4). Patients with dependent children indicated the greatest variety of responses, ranging from acceptance, satisfied, alright, overwhelmed, and irate. Only 11% (n = 5) of patients completed a third EJB assessment. Due to the small data size, there were no significant responses for ADI scores for patients with social determinants. 

Performance status scores were collected routinely based on clinical workflow. Of the patients who had ECOG scores, the median was 1 at each EJB assessment. The highest score seen was 2, indicating ECOG remained low regardless of EJB responses and time point collected. Similarly, NCCN-DT scores were collected during routine clinic care with medians of 0 or 1 at each EJB assessment. It is worth noting patients with higher NCCN-DT scores had correlating EJB selections, indicating consistency between the assessments.

Treatment was initiated prior to initial emotional assessment for 60% of patients, while 40% were treatment naive. Common baseline emotions were optimistic (47% vs. 36%, respectively), satisfied (11% vs. 25%), acceptance (11% vs. 11%), and overwhelmed (5% vs. 11%). Patients who did not initiate treatment also reported emotions such as angry, scared, and stressed. All EJB responses with corresponding treatment state are shown in Table 6.

## 4. Discussion

Current gaps in how information is provided to cancer patients suggest more personalization is needed to improve outcomes [6]. Recent data exploring digital interventions report reduced psychological distress and improved quality of life [3]. Systematic reviews have noted that clinician communication and written/visual information reviewed outside the consultation are associated with improvement. For example, systematic reviews of the teach-back method have noted improved learning-related outcomes such as recall/retention as well as medical outcomes such as re-admissions [8]. Despite the increased medical payoff for empowering patients with information they can understand, few studies have evaluated baseline LPs prior to a communication event. In fact, most of the only data in this space is embedded within science and pedagogy, with recommendations ranging from adoption of the VARK model of LPs to integration of multiple different learning process models [12,14]. Our project is the first to evaluate a patient co-designed LP tool to test feasibility of incorporation into a busy oncology setting with available online resources aligned accordingly.

Although clinicians deliver most clinical information verbally, LPB results revealed patients with PC had high preferences for linguistic and visual learning, not auditory. Secondary LPs were present in more than half of patients, implying LPs may be multi-modal. Performing the EJB assessment on patients not only revealed patient emotional states but also provided additional insight into the patient’s state of mind. At initial assessment, most patients experienced positive emotions of optimism, satisfaction, and acceptance. This was also seen across sub-cohorts of patients with high ADI scores and social determinants; however, there was a wider spectrum of emotions these patients faced.

### 4.1. Learning Preferences (LPs)

When comparing standard nursing assessment patient LPs, the LPB instrument more accurately assessed patient LPs than those subjectively assessed by nursing personnel, which were generic, and would better inform how patients should receive education. When presented with multiple modalities of educational materials, most patients accessed those aligned with their primary LPs. This further indicates the benefit for clinicians to ensure patients are receiving information in alignment with patient LP. In this project, 66% of patients accessed P2L. Older patients were not as likely to utilize P2L, likely based on lack of comfort with technology use or preference for the tangible C101 planner, signifying an opportunity to engage care partners in the future and/or assess digital literacy. P2L access codes were given to the patients; however, care partners may have accessed the platform if patients shared the login details. Future work to include access codes for care partners may prove beneficial to identify individual login characteristics.

### 4.2. Emotional States

Performing the EJB assessment on patients not only revealed patient emotional states but also provided additional insight into the patient’s state of mind. Although studies have shown less depression and increased positive attitudes with discussions by clinicians engaged in their emotional and social needs, most patients do not report these discussions taking place [17,18]. In our project, at initial assessment, most patients experienced positive emotions of optimism, satisfaction, and acceptance. This was seen across sub-cohorts of patients with high ADI scores and social determinants; however, there was a wider spectrum of emotions these patients faced. Incorporation of the LPB and EJB along the treatment trajectory thus allows for more tailored consultation experiences.

EJB assessments were in alignment with NCCN-DT scores; however, the specific selection of the emoji and verbal justification allowed for greater insight than the standardized scoring system. When considering the timing of initial assessment, there was a notable difference in EJB responses based on treatment timelines. Treatment naive patients experienced emotions of anger, being scared, and stressed, while patients already on treatment had more positive emotions. Improving the current practice paradigm by appropriately educating patients with neutral/positive emotional states may lead to better patient–clinician communication.

### 4.3. Shared Decision Making

These findings also have implications to improve the patient experience through enhanced shared decision making (SDM). This collaborative technique includes patient–clinician discussion of available treatment options, the risks/benefits of each, as well as the patient’s preferences. Ultimately patient–clinician partnering to determine the best course of action could be stronger if the clinician had knowledge of the individual LP and emotional state at the POC [19]. Since increasing patient engagement alleviates anxiety, depression, and fatigue, with reduced distress in both patients and their families, providing clinicians with these tools could allow them to personalize each visit and potentially lead to improved outcomes [20,21].

As one challenge with implementing SDM is health literacy, our results suggest that these tools may provide a way to improve disparities. Low health literacy can lead to confusion and can negatively impact decision-making, patient experience, and health outcomes. Further study is warranted to determine how much personalizing education at the POC can mitigate such differences and improve SDM. 

### 4.4. Project Implications

#### 4.4.1. Practical Implications

These findings have implications for improving the summary of the patient’s clinical encounter, especially given the 21st Century Cures Act which provides patients with real time data access. Providing a more detailed linguistic plan that patients/care partners can reference along the journey could alleviate decisional regret. There could be the potential for layering in visual references as well, such as graphic representation of the anatomy. This work has implications to be integrated not just in the management of patients with PC but also across other cancers.

#### 4.4.2. Conceptual Implications

Future research strategies include the additional emotional assessment of patients and care partners at different points of the care journey, with psychosocial support/interventions that would be deployed based on the corresponding emotional state. With the emerging efficiency potential of artificial intelligence (AI), this tool could be scored ahead of the clinician’s visit so that their entire experience including educational resources could be aligned with their learning preferences and emotional state. The integration of these results within the electronic health record can provide readily accessible personalization. This could help determine emotional readiness of the patient/care partner to engage in SDM. Tailoring education based on LPs offers a powerful avenue for enhancing comprehension and communication, which can enable patients/care partners to collaborate with their health care team. Although AI has tremendous benefit in allowing personalization prior to the clinic visit, patient engagement/interaction may be decreased due to lack of acceptability, trust, and interpersonal experience without including human interaction in the loop.

### 4.5. Limitations

There are several limitations inherent to this project. Firstly, not all patients were accessible to complete follow-up EJBs. This limited our ability to observe patient emotional changes throughout their treatment journey. In addition, emotional status can fluctuate within a short time frame based on extenuating factors, therefore EJB responses may have evolved depending on the timing of assessment. The project population was relatively homogeneous with respect to race/ethnicity. This raises the concern for potential sampling bias, as well as the scalability to more culturally diverse populations. Selection bias may have also had an impact on project results. Given that no baseline digital literacy assessment was performed, heterogeneity of patient age and digital literacy could have influenced a patient’s reluctance towards P2L engagement. In addition, no statistical analyses or formal tests were conducted to assess the validity or reliability of these measures. Future studies should use larger samples and conduct statistical tests to examine the validity and reliability of the LPB.

## 5. Conclusions

Personalizing patient education utilizing LPs and emotional states allows for an individualized approach to caring for PC patients with the potential for future exploration in other cancer disease sites. Providing targeted information prescriptions of curated resources adds the potential to address specific needs and support patients in understanding their condition to make informed decisions, facilitating a more supportive and empathetic relationship between patients and their healthcare teams. Additionally, care partner EJB assessments may prove beneficial in understanding the emotional burden associated with the phase of the patient’s care journey. Correlation with patient social determinants and ADI state scores may prove to be substantial given our findings; however, a larger cohort is needed to make conclusions. Future research in this area to evaluate how integration of such digital tools, with potential AI enhancements, can affect patient satisfaction, SDM, patient experience and health outcomes is warranted.

## Figures and Tables

**Table 1:** Patient Demographics.

Characteristic		Percent of Patients
*Social Determinants*	Lifestyle Concerns	30%
	Education Level of High School/GED or Trade School	29%
	Financial Concerns	20%
	Dependent Children in the Household	14%
	Insurance/Employment Disability	9%
*Insurance*	Medicare	60%
	Commercial	25%
	VA (veterans)	4%
	Out of Network or Not Insured	11%
*Regionalization*	Northwest Florida	2%
	Central West Florida	41%
	Central East Florida	19%
	Southwest Florida	30%
	Southeast Florida	4%
	Out of State	4%

**Table 2:** Multimodal Learning Preferences (Primary and Secondary).

Secondary Preference	Primary Learning Preference				
	*Visual (n = 16)*	*Linguistic (n = 21)*	*Auditory (n = 5)*	*Kinesthetic (n = 4)*	*Auditory/Visual Combination (n = 1)*
*Visual*	-	19%	0%	50%	-
		(n = 4)	(n = 0)	(n = 2)	
*Linguistic*	31% (n = 5)	-	40%	25%	0%
			(n = 2)	(n = 1)	(n = 0)
*Auditory*	19% (n = 3)	29%	-	25%	-
		(n = 6)		(n = 1)	
*Kinesthetic*	6% (n = 1)	-	0%	-	0%
			(n = 0)		(n = 0)

**Table 3:** Nursing Assessment Learning Preferences.

Nursing Assessment Learning Preference	Number of Patients	Primary Learning Preference from LPB
Demonstration, reading, video/visual	3% (n = 1/38)	Linguistic
Demonstration, reading, verbal	5% (n = 2/38)	Auditory = 1 Visual = 1
Demonstration, verbal, video/visual	3% (n = 1/38)	Visual
Reading, verbal	5% (n = 2/38)	Auditory = 1 Visual = 1
Reading, verbal, video/visual	76% (n = 29/38)	Auditory = 2 Kinesthetic = 3 Linguistic = 16 Visual = 7 Visual/Auditory = 1
Verbal	8% (n = 3/38)	Linguistic = 1 Visual = 2

**Table 4:** Prescription to Learn Access by Age.

Number of Resources Accesses	Age				Gender	
	*50–60 Years*	*61–70 Years*	*71–80 Years*	*81–90 Years*	*Male*	*Female*
	*(n = 4)*	*(n = 12)*	*(n = 10)*	*(n = 5)*		
*1 (n = 15)*	100%	42%	30%	60%	33%	67%
	(n = 4)	(n = 5)	(n = 3)	(n = 3)	(n = 5)	(n = 10)
*2 (n = 13)*	0%	50%	50%	40%	54%	46%
	(n = 0)	(n = 6)	(n = 5)	(n = 2)	(n = 7)	(n = 6)
*3 (n = 1)*	0%	0%	10%	0%	100%	0%
	(n = 0)	(n = 0)	(n = 1)	(n = 0)	(n = 1)	(n = 0)
*6 (n = 1)*	0%	8%	0%	0%	100%	0%
	(n = 0)	(n = 1)	(n = 0)	(n = 0)	(n = 1)	(n = 0)
*8 (n = 1)*	0%	0%	10%	0%	0%	100%
	(n = 0)	(n = 0)	(n = 1)	(n = 0)	(n = 0)	(n = 1)

**Table 5:** Emotional Journey Barometer Audio Recording Transcriptions.

Chosen Emotion	Transcribed Responses
Acceptance	I’m starting to feel more relaxed with the procedures as I go through them, [both] with the radiation and the other doctors that I’m going to meet to converse about my plan. So, I think things are heading in the right direction.
Satisfied	I chose “satisfied.” I felt that was the best [answer] because I came in confused, unsure, not knowing, and I got answers.
Overwhelmed	I am not overly distressed. I do have some grave concerns but basically [I have] a whole lot of information I have to sort through to digest.
Angry	I am angry, I am pissed off, because I didn’t do any of the [things] to get this, and I got it, and I’m as mad as I can be.
Scared	[The reason] why I’m scared is the unknown. I don’t know how long I’m going to live and what the pain is going to be like. I don’t know what else.
Stressed	I am feeling stressed because it’s all at once and it’s hitting me. I know how serious this is because I’m an RN and things can always go wrong. I’m afraid that I won’t make it through surgery.
Anxious	I’m worried about things I shouldn’t be worried about. Things that are coming up in my care. Anxious about the next chemo treatment, this one didn’t go very well.
Optimistic	I’m optimistic that this is going to work and give me a few more months.
Happy	I’m happy because I have a plan and I’m going to beat this.

**Table 6:** Emotional Journey Barometer by Treatment.

EJB Assessment	Emoji Response	Treatment State
*Assessment 1* *(n = 47)*	Acceptance	40% treatment naive
		20% on chemotherapy
		20% pre radiation
		20% on radiation
	Acceptance/Optimistic	100% treatment naive
	Angry	100% treatment naive
	Anxious	50% on chemotherapy
		50% pre radiation
	Confused	100% on chemotherapy
	Happy	34% treatment naive
		33% on chemotherapy
		33% pre radiation
	Optimistic	40% treatment naive
		50% on chemotherapy
		5% pre radiation
		5% on radiation
	Overwhelmed	34% treatment naive
		33% pre radiation
		33% on radiation
	Satisfied	33% treatment naive
		45% on chemotherapy
		22% pre radiation
	Scared	100% treatment naive
	Stress	100% treatment naive
*Assessment 2* *(n = 21)*	Acceptance	14% treatment naive
		14% pre radiation
		72% on radiation
	Alright	100% on chemotherapy
	Happy	100% post radiation
	Irate	100% on chemotherapy
	Optimistic	34% treatment naive
		14% on chemotherapy
		29% on radiation
		14% post radiation
	Overwhelmed	100% on radiation
	Satisfied	50% on chemotherapy
		50% on radiation
*Assessment 3* *(n = 5)*	Acceptance	100% on radiation
	Confused	100% treatment naive
	Nervous	100% on radiation
	Optimistic	100% pre radiation
	Satisfied	100% treatment naive
*Assessment 4* *(n = 1)*	Happy	100% post radiation

**Figure A1:**
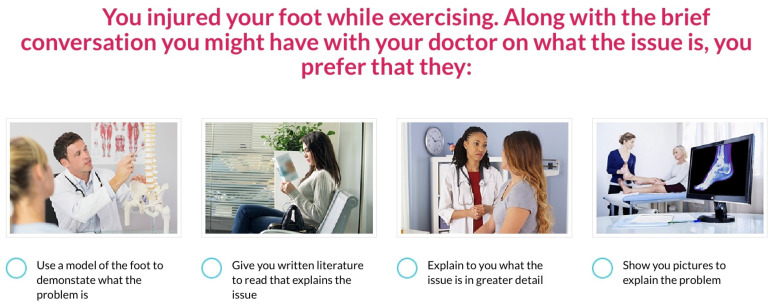
Sample LPB question.

**Figure A2:**
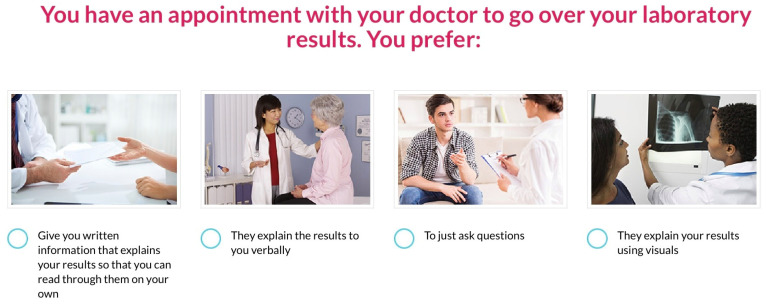
Sample LPB question.

**Figure A3:**
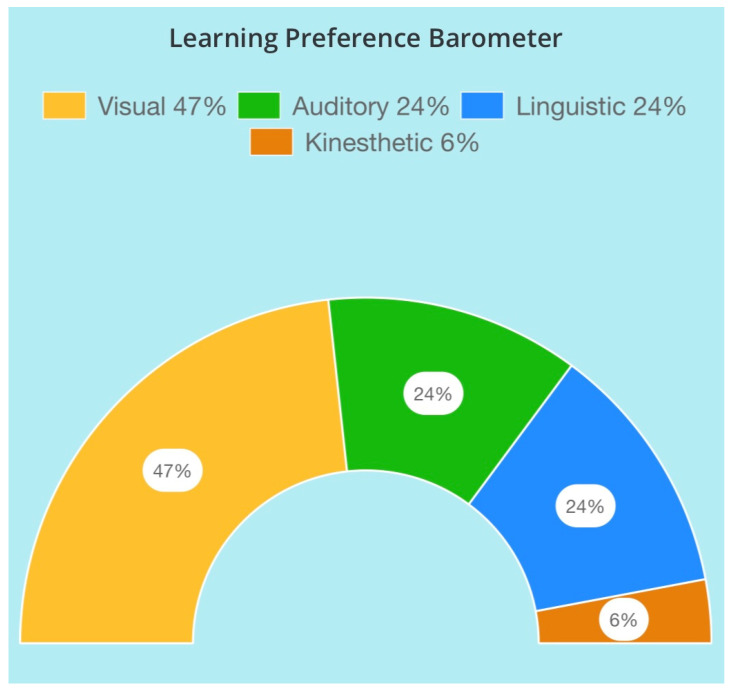
Sample LPB result.

**Figure A4:**
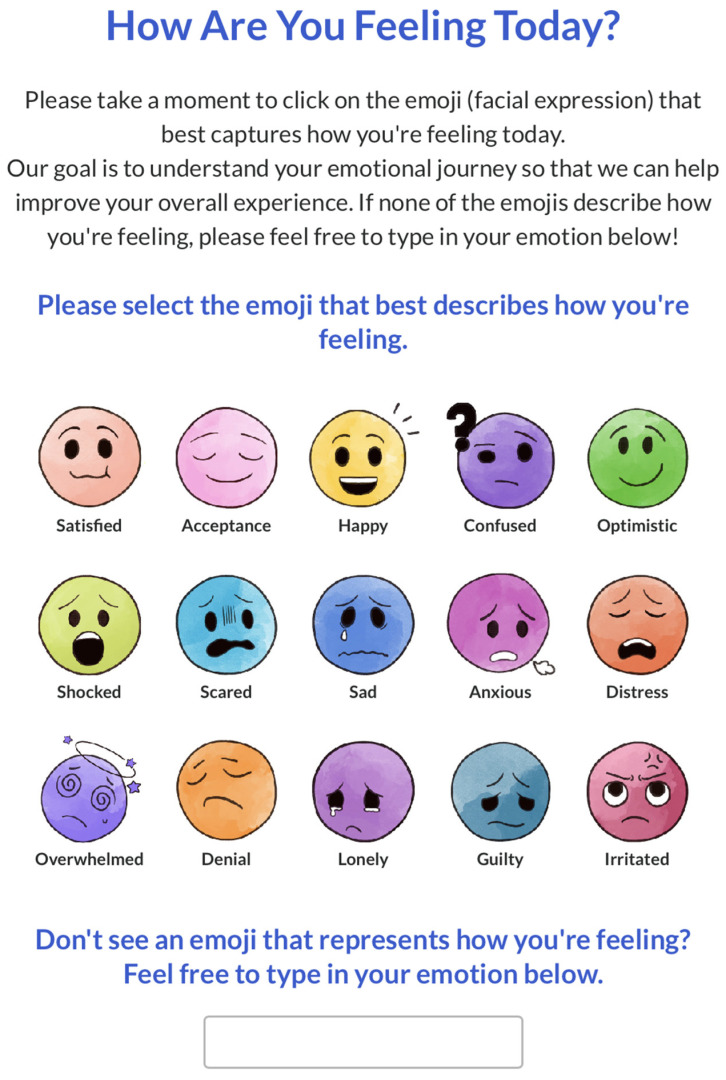
Illustration of EJB tool.

**Figure A5:**
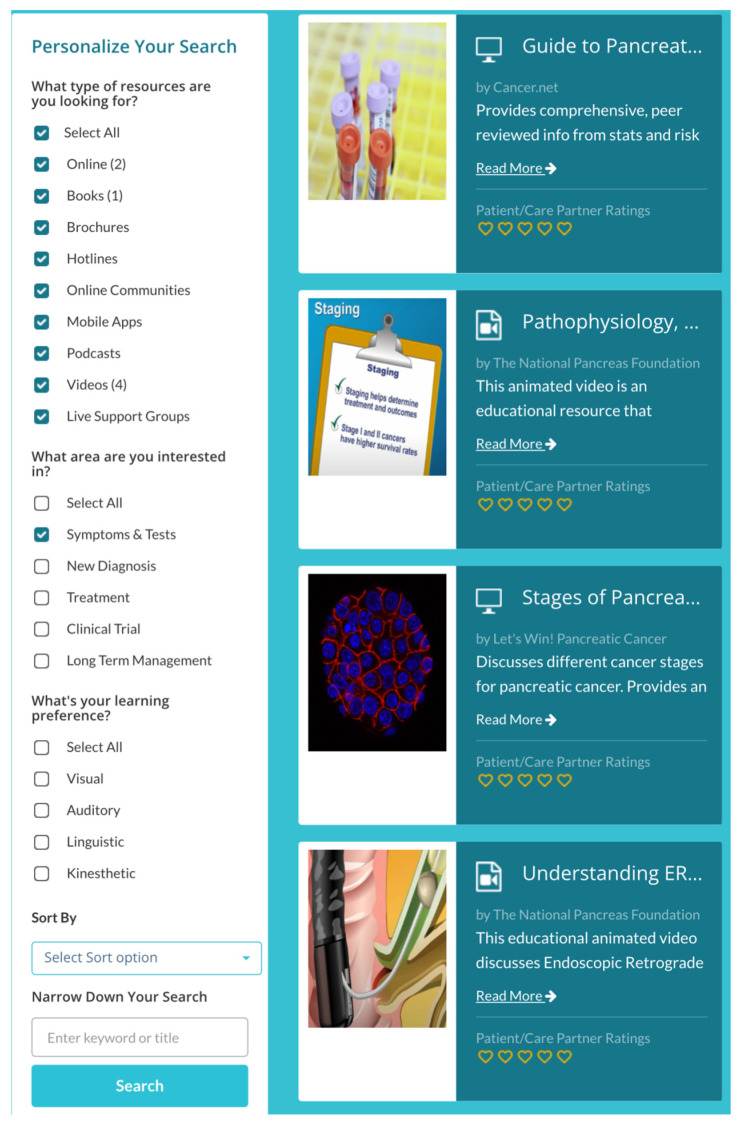
Illustration of P2L platform.

## Data Availability

The raw data supporting the conclusions of this article will be made available by the authors on request.

## Abbreviations

The following abbreviations are used in this manuscript:

PC	Pancreatic cancer
LPB	Learning preference barometer
EJB	Emotional journey barometer
C101	CANCER101
LP	Learning preference
P2L	Prescription to Learn^®^
POC	Point of care
CCI	Charlson Comorbidity Index
ADI	Area Depravation Index
ECOG	Eastern Cooperative Oncology Group
NCCN-DT	National Comprehensive Cancer Network Distress Thermometer
SDM	Shared decision making
AI	Artificial Intelligence

## Appendix A

### Appendix A.1. Learning Preference Barometer (LPB)

A participatory co-design process was utilized to partner with 322 patients and care partners to ensure that the LPB final instrument aligned with their needs, preferences, and individual experiences. A landscape assessment was conducted to review various surveys and processes used to understand learning preferences. These methods were then evaluated by patients across various domains, including patient-centricity/user experience, motivation to use/engagement and context, impact, and relevance to healthcare. Free form feedback was also provided to understand specific needs. Based on these insights, the LPB was co-designed in collaboration with these patients and care partners. We then tested the LPB against other validated/reliable tools (VARK Questionnaire) to ensure that the final output was consistent. A separate group of patients and care partners was then given access to the LPB, which was assessed using a structured scorecard to ensure satisfactory patient/care partner usability and acceptability was reported.

The LPB can be accessed at: https://www.prescription2learn.org/learning-preference-barometer, accessed on 4 August 2025. Sample questions from the LPB are included below (Figure A1 and Figure A2). Note that each response correlates to a specific learning preference; i.e., the first response relates to kinesthetic, second to linguistic, third to auditory, and fourth to visual in the first example.

### Appendix A.2. Emotional Journey Barometer (EJB)

The EJB is a tool that was co-designed with 94 patients/care partners to capture the main sentiments they experience during their health journey. Emojis provide a simple method of conveying emotions and mood with the benefit that the concept of emojis is well integrated into modern daily life through the prevalent use of digital devices and social media. This method helps fill in cues otherwise missing from alternate forms of communication that can be more difficult to describe and helps document quantitative data that can be acted upon. Neutral color tones were used given the controversy around lack of representation in existing emojis and color tones currently available on social media.

To initially map the most prevalent emotions experienced, qualitative semi-structured interviews were conducted utilizing a conversational, narrative, ethnographic approach to allow patients/care partners to piece together and share their health stories in a meaningful way and provide an opportunity for the co-production of journey maps. A participatory storytelling technique was used to provide a safe zone for information exchange with a deeper dive into the emotions experienced and the rationale across phases of the care journey. Based on pre-defined phases of the patient pathway, the main emotions experienced by patients/care partners were then separately plotted on a graph based on story sharing and responses to various questions. 32 different emotions were expressed and individual mapping across the patient pathway was shared with patients and care partners for confirmation. 11 emotions were outliers experienced by less than 3 patients/care partners. The remaining 21 emotions were the most prevalent emotions experienced and used to co-design the EJB. Various emoji options were provided to patients/care partners for each of the 21 emotions to allow them to choose the most aligned with opportunity to provide additional feedback as emojis were further revised. Final emojis were validated with patients/care partners without labels to ensure there was no confusion and ensure the emoji matched the emotion it was intended to convey. Labels were then added to each emoji to further describe the sentiment to mitigate any ambiguity. We then confirmed the relevance of the 21 emotions captured in the EJB with 88 different patients/care partners across different points in their health journey with the opportunity to express free form emotions that were not depicted with emojis. Patients/care partners then further described the emotion selected to ensure that the sentiment experienced matched the sentiment expressed through the emoji. No deficiencies were identified. In addition, patients/care partners were asked to assess the EJB using a structured scorecard to ensure satisfactory usability and acceptability was reported.

A subset of 15 emojis was selected for this project; we removed the following emojis (Indifferent, Skeptical, Disappointed, Curious, Hopeless, Strong) out of the original 21 to simplify based on the most prevalent emotions experienced in the cancer setting. The below sample illustrates the emoji format and open-ended response box (Figure A4). Note that emoji names are depicted to ensure patients/care partners associated the emoji with the correct label.

### Appendix A.3. Prescription to Learn^®^

P2L was co-designed with 125 patients/care partners and 22 clinicians, as well as academic medical centers and advocacy partners using a rigorous curation and review process to map the most effective resources from credible sources and co-produce all screens in the system. Patients and care partners can filter through curated educational resources by their disease state, phase in the journey, medium of choice and learning preference. Clinicians can also prescribe resources to their patients.

This project included access to a white-label P2L platform that contained specific resources selected by our participating institution (Figure A5). 25 resources were curated to include topics of symptoms and tests, new diagnosis, treatment, clinical trials, and long-term management. Resources were a blend of institutional and open-source educational materials that were tagged with corresponding learning preference(s). Filters were included to enhance the search experience for patients/care partners and offer the opportunity for personalization of learning preferences and medium of choice.

### Appendix A.4. Area Depravation Index (ADI)

As stated, LPB and EJB assessments were reviewed in relation to ADI scores. See the below table for further details (Table A1). Note that patients with higher ADI scores more often were identified as having visual LP. When considering the EJB results, sample sizes limit the capability to draw conclusions. However, the baseline EJB indicated that patients with higher ADI scores tended towards optimistic and satisfied emojis. In addition, assessment 2 results showed patients with higher ADI scores tended towards the acceptance emoji.

**Table A1:** ADI scores in relation to learning preference and emoji responses.

Characteristic		ADI Score Median (Range)	
*Learning Preference*	Auditory (n = 5)	6 (4–10)	
	Kinesthetic (n = 4)	5 (2–7)	
	Linguistic (n = 21)	6 (1–10)	
	Visual (n = 16)	8 (2–10)	
	Visual/Auditory (n = 1)	5	
		Baseline ADI	Assessment 2 ADI
*Emoji Responses*	Acceptance	5 (1–8) (n = 5)	8 (1–9) (n = 7)
	Acceptance/Optimistic	2 (n = 1)	-
	Alright	-	4 (n = 1)
	Angry	8 (n = 1)	-
	Anxious	6.5 (4–6) (n = 2)	-
	Confused	9 (n = 1)	-
	Happy	9 (2–10) (n = 3)	7.5 (5–10) (n = 2)
	Irate	-	2 (n = 1)
	Optimistic	7 (2–10) (n = 20)	5 (2–10) (n = 7)
	Overwhelmed	3 (2–6) (n = 3)	3 (n = 1)
	Satisfied	8 (3–10) (n = 9)	6 (5–7) (n = 2)
	Scared	9 (n = 1)	-
	Stress	2 (n = 1)	-
